# Geopolymers and Functionalization Strategies for the Development of Sustainable Materials in Construction Industry and Cultural Heritage Applications: A Review

**DOI:** 10.3390/ma15051725

**Published:** 2022-02-25

**Authors:** Fausta Giacobello, Ileana Ielo, Hossem Belhamdi, Maria Rosaria Plutino

**Affiliations:** 1 Institute for the Study of Nanostructured Materials, ISMN–CNR, Palermo, c/o Department of ChiBioFarAm, University of Messina, Viale F. Stagno d’Alcontres 31, Vill. S. Agata, 98166 Messina, Italy; fausta.giacobello@ismn.cnr.it (F.G.); ileana.ielo@ismn.cnr.it (I.I.); 2 Department of Engineering, University of Messina, Contrada di Dio, S. Agata, 98166 Messina, Italy; hossem.belhamdi@unime.it

**Keywords:** hybrid materials, geopolymers, aluminum silicates, cultural heritage

## Abstract

In the last decades, new synthetic hybrid materials, with an inorganic and organic nature, have been developed to promote their application as protective coatings and/or structural consolidants for several substrates in the construction industry and cultural heritage field. In this context, the scientific community paid attention to geopolymers and their new hybrid functional derivatives to design and develop innovative and sustainable composites with better chemical resistance, durability and mechanical characteristics. This review offers an overview of the latest progress in geopolymer-based hybrid nanofunctional materials and their use to treat and restore cultural heritage, as well as their employment in the building and architectural engineering field. In addition, it discusses the influence of some parameters, such as the chemical and physical characteristics of the substrates, the dosage of the alkaline activator, and the curing treatment, which affect their synthesis and performance.

## 1. Introduction

The preservation and restoration of cultural heritage is considered by modern society to be a great responsibility toward future generations. This is due to the continuous damage and deterioration of artworks and monuments caused by the increasing air pollution in industrialized areas [1]. Although many monuments and works of art require minimal restoration interventions, most historic buildings and archaeological finds show conservation problems that need primary renovation operations. In the current practice, the restoration processes are guided by preliminary diagnostic analyses on the artworks under study, in order to discover materials with characteristics as similar as possible to those of the historical finds [2,3]. Compatible products are chosen based on their comparable physico-chemical and mechanical properties and their similar aesthetic characteristics. Moreover, other different factors can influence the choice of the restoration material, such as thermal resistance, adequate ductility, low creeping and penetration ability, in addition to suitable mechanical properties to decrease the risk of collapse phenomena [4]. In the last decades, new synthetic materials have been developed to promote their application as structural consolidants and protective coatings for different substrates, such as ceramic, wood, steel, concrete walls, textiles, and paintings. Furthermore, these inorganic or organic materials are used to restore historical and cultural interest assets, as well as being employed in the modern and sustainable building and architectural engineering sector [5]. In this scenario, the attention toward geopolymers arises from the interest in creating and developing innovative materials with better chemical resistance, and mechanical and durability properties [6]. Geopolymers are aluminosilicate-based amorphous inorganic materials obtained through a polymerization process, starting from natural or waste materials with a high content of aluminum or silicon, such as slag from blast furnaces from steel mills, clays, flying and volcanic ashes or other aluminosilicate sources, such as laterite deriving from tropical areas, which offers acceptable mechanical properties to geopolymerization products [7,8,9,10,11]. The reaction of the precursor powder with an activating alkaline solution, consisting of sodium and/or potassium hydroxides and silicates, at a temperature below 100 °C, produces this alkali-activated material, which is the amorphous analog of the zeolite. Geopolymer studies, nowadays, have considerably grown since their extensive use in many applications and industrial fields [7,8,12]. These applications are found in the automotive and aerospace industries, non-ferrous foundries and metallurgy, civil engineering, cements and concretes, ceramics and plastics industries, waste management, retrofit of buildings, and, as already mentioned, in art and decoration [13]. As concerns their use as cement and concretes, the main present challenge is to produce new structural materials or optimize the existent ones with reducing consumption during production, the emission of greenhouse gases and environmental impact [7,14]. Moreover, ordinary concretes show disadvantages, such as low thermal and fire endurance, little chemical resistance against acids and salt and significant worldwide carbon dioxide emissions [15]. Therefore, as a source of innovation in the engineering field, alternative construction materials are constantly being developed and tested by the academic and scientific community.

According to Provis and van Deventer [16], Pudron was the first to show the synthesis of building materials using alkaline activation starting from high-calcium blast furnace slags. However, the interest in this new technology began to mature in the 1950s when Glukhovsky demonstrated, through an investigation on ancient Roman and Egyptian structures, the possibility of preparing new materials through the reaction of aluminosilicate raw materials with solutions containing alkaline compounds, such as carbonates, hydroxides, and silicates [17]. However, geopolymers were developed only in 1976 with the findings of the French engineer Joseph Davidovits, who patented several aluminosilicate formulations [13,18].

In 1997, Krivenko named these binders “geocements”, since the formation and features of their hydration products were similar to those of some natural minerals [19]. From the first Davidovits patent to the present day, the interest in these materials has greatly grown, especially since the early 21st century, with a relative increase in scientific publications and conferences on this topic. Several alkaline activation studies, currently reported in the literature, deal with the chemical reaction between a solid aluminosilicate precursor and an alkaline activator, at room temperature, resulting in a robust composite [20]. These materials can be used in high-performance concretes and in ceramics strengthened with fibers, with features that are comparable or even higher than ones related to the conventional Portland cement or other composite materials produced by traditional techniques. Granulated blast furnace slag (from the steel industry), fly ash (from coal combustion) and natural clays (metakaolin and haloysite or kaolinite fired at 700 °C), since their low cost and availability, are employed as main precursors for producing alkali-activated materials [21,22,23,24], even if their production process allows to obtain a non-homogeneous products, which could cause problems in the geopolymer synthesis [16]. At this time, volcanic tuffs are the best materials for producing geopolymer cement [16,25]. Another problem linked to the synthesis is the alkali activation of the alumina–silicate precursors, whose process was not fully described yet. As previously mentioned, alkali-activated materials have shown excellent eligibility to preserve and restore ancient and modern constructions. According to their reaction conditions and composition, especially precursors, these materials can be versatile and easily adaptable on site [26]. Nevertheless, geopolymers are featured by scarce rheological properties due to their low viscosity, making them almost inapplicable in restoration works mostly of vertical buildings [9,27]. To overcome this problem, researchers have recently tried to improve the chemical, physical and mechanical properties of these compounds by formulating hybrid organic–inorganic systems. These new binders, consisting of geopolymers with an organic component, are synthesized through a cross-link reaction between the inorganic phase and the organic one, such as polysiloxane oligomers, alkoxysilane agents or epoxy resin precursors [5,28,29]. The functionalization of the alkali-activated material with the organic component improves the system’s performance, as it allows an increase in the mixture’s viscosity, making the hybrid material more versatile in architectural and artistic restoration [28,30]. The synthesis process of these hybrid materials occurs via the sol–gel method, which is generally employed to produce a wide variety of materials, from inorganic glasses to organic–inorganic compounds, such as the ones described above [5,30]. Through this method, which allows producing multi-component materials, it is possible to obtain compounds that show peculiarities deriving from the synergy of the two components having different nature, rather than from the addition of individual contributions [5,28].

This review offers a recent overview of the following:

- Chemistry of geopolymers, focusing on the factors affecting the reaction mechanism of the polymerization process;
- The development of new hybrid materials deriving from functionalization with organic and inorganic nanomaterials or polymers;
- Their possible applications in the cultural heritage sector through a critical analysis of the various data present in the literature.

## 2. Geopolymers Chemistry and Reaction Mechanism

**Figure 1:**
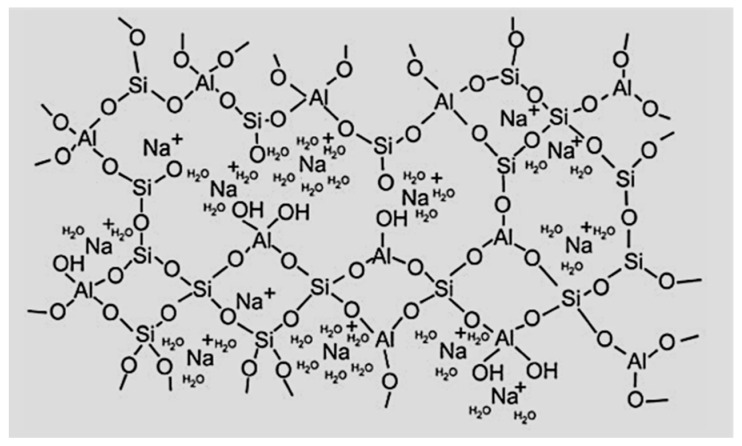
Schematic model of the geopolymer structure based on the assertions of Barbosa et al. and Rowels et al. [32,33].

Geopolymers, as already mentioned, are one of the potential substitutes for cement-based composites. These are ceramic-like inorganic polymers, defined as synthetic inorganic aluminosilicate-based polymers, obtained from a reactive powder, such as metakaolin or fly ash, source of silicon and aluminum, and an activating alkaline solution, such as sodium or potassium hydroxide [16]. Their chemical composition is similar to natural zeolitic materials and the microstructure consists of chains or networks of inorganic molecules composed of one silicon or aluminum atom connected through covalent bonds to four oxygen atoms and forming tetrahedrons linked to each other in a three-dimensional network with one common oxygen atom [31]. Several attempts were conducted to draw schematic diagrams of the three-dimensional geopolymer structure, because of their complex arrangement proposed several years ago by Davidovits [13]. Further investigations on the microstructure of the alkali-activated products use different methods, such as thermal analysis, mercury porosimetry, BET isothermal curves, and MAS NMR spectroscopy (Si, Al and especially Na) allowed Barbosa et al. to make some revisions of the framework [32]. However, the most helpful picture of the ones currently available was recently proposed by Rowels et al., and it is shown in Figure 1 [33]. 

**Figure 2:**
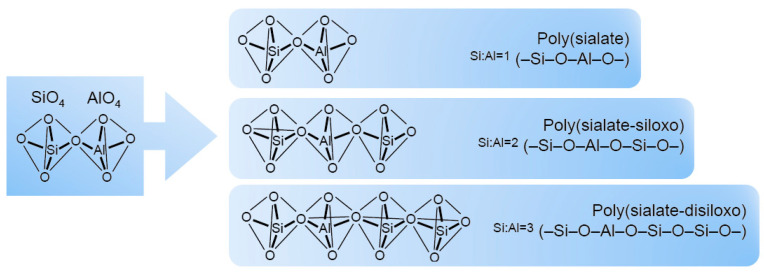
Representation of oligomeric units of geopolymers according to the Davidovits model [13].

Several years ago, Davidovits introduced the ‘sialate’ nomenclature to define aluminosilicate structures. Si–O–Al connections were described as sialate bonds and the Si–O–Si ones were named siloxo bonds. Through this classification, it was possible to obtain a method to describe the composition of geopolymers based on their Si/Al ratio [13,16]. The most common oligomeric units, represented in Figure 2, are: –Si–O–Al–O– sialate, poly(sialate); –Si–O–Al–O–Si–O– sialate-siloxo, poly(sialate-siloxo); –Si–O–Al–O–Si–O–Si–O– sialate-disiloxo, poly(sialatedisiloxo) [8].

**Figure 3:**
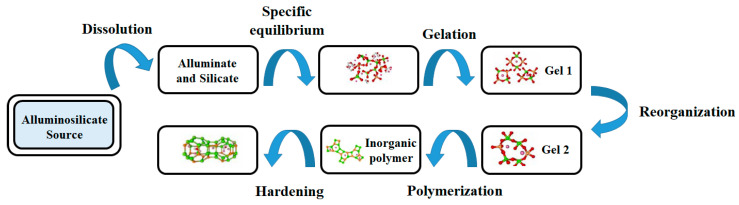
Schematic representation of the geopolymerization mechanism.

Moreover, geopolymers materials show structural similarities to zeolitic materials and vitreous bodies, exhibiting the same three-dimensional arrangement, but with some differences, such as the presence of the interstitial water and a porous structure [16,34]. The general reaction mechanism, already described by Glukhovsky et al. in 1950, also known as geosynthesis or geopolymerization, is characterized by a multistep process, shown in Figure 3.

During the geopolymerization process, an amorphous or semi-crystalline polymeric resin is formed. It acts as an adhesive for the aluminosilicate-based raw materials that have not reacted or for any fillers that make the material functional, improving the chemical–physical or mechanical properties depending on the applications for which the finished products are intended. Essential variables in the polysialates production, proper to modify some features according to their use, are the Si/Al, M_2_O/Al_2_O_3_ and H_2_O/M_2_O ratios, where M refers to monovalent alkali metal cations, such as Na^+^ or K^+^, the type of the cation inserted in the structure as well as the curing conditions, such as time and temperature.

### Assessment of Factors Affecting the Geopolymerization Process

Although the alkali activation of the alumina–silicate precursors has not been fully described yet, several papers and books in the literature report the possible mechanism of the process [13,16,17,35,36,37]. Strong alkaline solutions are required for the activation of Si and Al present in the reactive powder derived from raw material, such as granulated blast furnace slag (from the steel industry), fly ash (from coal combustion) and natural clays [23]. Generally, the activators used for the preparation of concretes and geopolymer binders are alkaline salts-based materials. Glukhovsky et al. classified them into six groups according to their chemical composition [38]. More in detail, these are divided into caustic alkalis, MOH, salts of weak acids (not silicates), such as M_2_CO_3_ or M_2_SO_3_, silicates M_2_O·nSiO_2_, aluminates M_2_O·nAl_2_O_3_, aluminosilicates, M_2_O Al_2_O_3_·(2–6) SiO_2_, and salt of strong acid (not silicates), such as M_2_SO_4_ [39]. Due to their wide availability and low cost, the common activators used are NaOH, Na_2_SO_4_, sodium and/or potassium silicate, Na_2_CO_3_, K_2_CO_3_, KOH and K_2_SO_4_ [19]. The choice among these depends on the nature of the precursors and the geopolymer features to be obtained. For example, silicate solutions increase the Si/Al ratio in the reactive powder and reach the value desired to obtain a product having specific properties. Hydroxides, instead, are generally used to modify the M_2_O/Al_2_O_3_ and M_2_O/H_2_O ratios, very important to balance the negative charge on the Al tetrahedra and to bring the pH to the values required for the dissolution of the alumina–silicate precursor at the beginning of the synthesis process [16,36,39]. Most of the time, both alkaline reagents are employed in the geopolymerization since they play a key role in the development of mechanical properties of the geopolymers [36]. The choice to combine both reagents is derived from the following reasons: the sodium silicate surplus inhibits water evaporation. It causes a decrease in the compressive strength of the geopolymer product. At the same time, an excess of OH^−^ ions leads to a reduction in the material resistance [36,40]. It is well known that the reaction of aluminosilicate materials in a strongly alkaline environment causes a breakdown of Si–O–Si and Al–O–Al bonds and forms a new phase, where Al atoms are inserted between the silicon and oxygen ones, breaking the covalent bond Si–O–Si that links them. 

Amorphous alumina–silicates are desegregated into monosilicate and aluminate species, as shown by the following Equation (1) [20,41]:

SiO_2_·Al_2_O_3_ + 4OH^−^ + 3H_2_O → [SiO_2_(OH)_2_]^2−^ + 2[Al(OH)_4_]^−^ (1)

[SiO_2_(OH)_2_]^2−^ + [Al(OH)_4_]^−^ → [(OH)O_2_SiOAl(OH)_3_]^3−^ + H_2_O (2)

Under the action of alkalis, the dissolved monosilicate and aluminate species condense (Equation (2)) and form an alumino-silicate gel, which can be regarded as a zeolite and geopolymer (see Figure 1) precursor through a final polycondensation step [16,42,43]. 

Positive ions present in the structure cavities, such as Na^+^, K^+^, Li^+^, Ca^2+^, Ba^2+^, NH_4_^+^ and H_3_O^+^, balance the negative charge of aluminum cation placed in tetrahedral coordination [19]. The general formula can described their composition: M_n_[-(SiO_2_)_z_-Al_2_O_3_]_n_ wH_2_O. Here, M is a cation, such as Na^+^, K^+^ and Ca^2+^, n is the degree of polycondensation, and z is 1,2, or 3 [19,34]. 

Different products, such as amorphous, partially amorphous and crystalline materials, can be formed [34,37]. The reagent concentrations play a significant role in the alkali activation process, allowing crystalline zeolite-type products for diluted suspensions and amorphous materials for high solid phase concentration [34,36]. The reactions of dissolution and formation of the products are strictly dependent on the pH values of the solutions and chemical compositions of the precursor materials [19,37,42,44]. The nature and the concentration of the alkali activators, as already mentioned, and the characteristics of the aluminosilicate precursor and curing temperature have the most important effects on the SiO_2_/Al_2_O_3_ ratio of the hydration products [36,37]. In many scientific articles, the microstructure of the hydrated products obtained from metakaolin, fly ash and furnace slags have been investigated through different analytical techniques, such as NMR, XRD, IR and SEM, which highlight an amorphous aluminosilicate gel as the main reaction product when it is performed at room temperature [19].

Alkali-activated fly ash cement show different microstructures if treated with different alkali activators [22]. Sodium hydroxide solutions allow for a very porous material in fly ash precursors, and the Si/Al together with Na/Al ratios noticed in the hydration products are 1.5 and 0.48, respectively [19].

Microporous materials, instead, occur when NaOH and Na_2_SiO_3_ are both used in the alkali activation, which fills the cavity between fly ash particles. In this case, the product, having Si/Al = 2.8 and Na/Al = 0.46 molar ratios, also shows some traces of mullite and particles with a high proportion of aluminum and iron [19,36].

Nevertheless, when potassium hydroxide is used as an alkaline activator, the molar ratios are Si/Al = 2.6 and K/Al = 1.55, respectively. This denotes an increase in the Si/Al ratio when the alkaline activator mixes NaOH and sodium silicate. In contrast, the Na/Al ratio remains constant and independent of the activator employed in the geopolymerization process [19]. Moreover, an increase of the Si/Al ratio and a decrease of the alkali concentration leads to the formation of a less crystalline product, and the presence of soluble silicates in alkali solutions improves the strength of the material, which results in weakening if treated with alkali containing little or no soluble silicates [19]. Another critical point in the geopolymer synthesis is the influence of the metal cation of the alkaline solution on the strength and hardening of the material produced by the alkaline activation [42]. It was noticed that potassium-based activators lead to a higher strength than sodium-based ones [42,45]. Moreover, when the SiO_2_/K_2_O ratio is lower in the reactive alkaline solution, it is notable an improvement in the geopolymerization [42]. In addition, the cation size influences the morphology of the material particles: the Na^+^ ions, having a smaller dimension than the K^+^ ions one, promote the formation of monomer silicates, whilst the larger size of K^+^ cations encourages the formation of a large oligomer, having greater reactivity with the Al(OH)_4_^−^ species. Therefore, geopolymers produced using KOH solutions have higher compressive strength than those synthesized using sodium hydroxide. Activator efficiency also depends severely on the pH value, which governs the initial dissolution of the precursor material and subsequent condensation reactions [36,46]. Indeed, it is well known that the initial pH values of the reactive solution and the addition of other chemical agents can influence the properties of the alkali-activated products. Many papers highlight that the activation of the raw materials depends strictly on the initial pH of the activator solutions, and the rate of the hydration reaction is increased for higher pH values [43,47,48]. NaOH solutions lead to an extreme alkaline environment (higher than any other sodium-containing compound), short geopolymerization time and high hydration rate [19].

However, the pH is not the only critical point. Additionally, the type of reagent can influence the progress of the reaction. For example, although the pH of the Na_2_SiO_3_ solution is higher than the one of Na_2_CO_3_ activated material, the induced reaction for this alkaline reagent is higher, and the hydration rate lower than the latter [19,36]. It seems that the hydration characteristics of alkali-activated materials, especially slag, are more influenced by the nature of anions of the activator than the initial pH of the solution [19,49]. Moreover, the addition of chloride salts can cause a significant adverse effect on the hardness of geopolymer material, as in fly ash alkali-activated products [48]. Therefore, to increase the material’s strength and improve its characteristics, it is necessary to consider some parameters, such as the chemical characteristics of the substrates, the curing treatment and the dosage of the alkaline activator [36].

**Figure 4:**
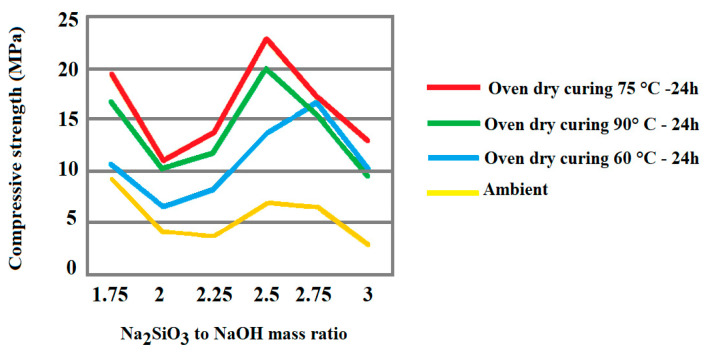
Effect of sodium silicate/sodium hydroxide mass ratio for ambient and oven dry-curing [50].

A study published by Joshi and Kadu dealt with the effect of the variation of mass ratio on the compressive strength for ambient and ordinary curing, in alkaline activation of eco-friendly fly ash [50]. As shown in Figure 4, the ratio of 2.5 provides the best possible strength at all temperatures. Maximum compressive strength is observed in oven-dry curing at 75 °C for 24 h.

**Figure 5:**
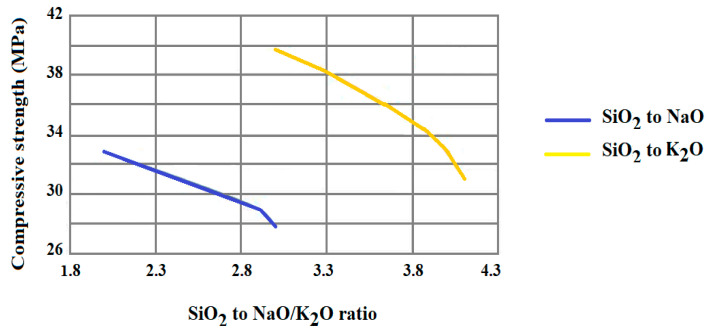
Compressive strength of a geopolymer fly ash based vs. SiO_2_ to Na_2_O/K_2_O mass ratio [42].

Another example of how the chemical nature of the precursors and the alkaline reagents used in the synthesis of geopolymers can influence the strength of the material produced was reported by Błaszczyński, T. Z. and Król, M. R. [42]. As shown in Figure 5, the hardness increases with the increase in the precursor and alkaline concentration. This is a consequence of the faster dissolution of (SiO_4_)^4−^ and (AlO_4_)^5−^ ions from fly ash and the rapid formation of sodium and potassium alumino-silicate, which lead to an increase in strength [42].

Another significantly important factor, which affects the geopolymerization process, is efflorescence. In the literature, it is well known that alkaline activators in a geopolymeric matrix can cause this phenomenon, indicating the accumulation of white salt crystals on porous surfaces of the constructions. For example, they sometimes appear on block walls or the surfaces of concrete structures, plaster, stone or brick, or on the joints of waterproof floors, such as ceramic. This event is related to free water presence in the mortar and hydroxides inside the matrix, used as alkaline activators. It appears as a uniform discoloration or as a localized surface deposit in the points where the water leaves the material. The chemical reactions that dominate this process are the following:

Ca(OH)_2 (aq)_ + CO_2 (g)_ → CaCO_3 (s)_ + H_2_O _(l)_ (3)

2NaOH _(aq)_ + CO_2 (g)_ → Na_2_CO_3 (s)_ + H_2_O _(l)_ (4)

**Figure 6:**
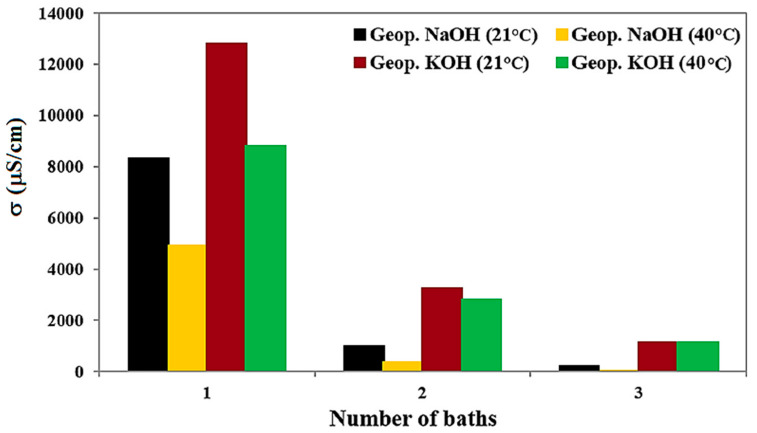
Electrical conductivity investigations on geopolymeric matrix treated with different alkali activators and different curing temperatures.

These reactions indicate the precipitation of sodium carbonates due to the reaction of the excess hydroxide (not reacted in the material) with carbon dioxide present in the atmosphere. Generally, efflorescence occurs for several reasons, such as the lack of compactness of the concrete, insufficient curing and atmospheric conditions that cause rapid drying of the cementitious material [51,52]. For example, Geraldes et al. in a study on the conservation of tiles, observed a higher amount of efflorescence in the pastes containing unreacted NaOH than the ones obtained using KOH [6]. This result is probably due to the cation size. It was assumed that Na^+^ is transported more easily on the material surface since its smaller dimension concerning K^+^. On the contrary, the larger latter remains trapped in the internal porous structure, leading to inner efflorescence phenomena. Therefore, the presence of soluble salts into historical ceramic and porous materials, such as tiles, mortars and stones, is undoubtedly detrimental. For this reason, it is necessary to reduce the amount of these compounds through a desalination process. Therefore, Geraldes et al. studied a possible method of salt removal and performed various investigations that consisted of immersing the inorganic matrix obtained by using different alkali activators in a water bath, monitoring its electrical conductivity [6]. The results of this research are reported in Figure 6. It is possible to note that the best result was obtained using NaOH as an alkaline activator and drying the inorganic matrix at T = 40 °C.

## 3. Synthesis of Functional Geopolymeric Hybrid Materials

For over 30 years, the study of organic–inorganic hybrid materials has aroused enormous interest in academia and industry. The synthesis mechanism can be described as polymerization or insertion into the inorganic matrix of nanoparticles or organic molecules, such as alkoxides, polysiloxanes and, more generally, polymeric precursors that can lead to a polycondensation [53]. The great attention in these multifunctional hybrid materials is associated with their peculiar chemical and physical properties deriving from the synergistic interaction between the organic and inorganic moiety, which does not depend on the simple sum of the individual contributions, but on the formation of a broad hybrid interface [53,54]. These organic–inorganic systems play a crucial role in developing and improving several features for the newly created material, such as mechanical, optical, chemical and thermal properties. These complex systems can be divided into two groups based on the interface nature, named class I and class II materials, respectively. The first ones are characterized by weak interactions between the components, such as Van der Waals forces and hydrogen bonds. The second ones are featured by strong interactions at the interface with covalent or ionic bonds. These hybrid materials can be processed employing several engineering methodologies, such as spin coating, dip coating, micro-emulsion, extrusion, aerosols and printings, which can lead to complex systems with different forms (thin films, foams, fibers, powders and monoliths), based on the type of use that they undergo. The synthesis of these complex systems can follow different routes: (i) the intercalation of an organic precursor into an inorganic matrix previously formed (e.g., insertion in clays or aluminosilicates, such as geopolymers or the handling of the inorganic compound, after drying, by applying a sol–gel dispersion on the monolith surface); (ii) the dispersion of an inorganic compound in a polymeric matrix; and (iii) the concurrent polymerizations of the inorganic and organic phase, which lead to a co-reticulation process with the formation of a hybrid network (e.g., the addition of alkoxysilane reagents in a geopolymeric matrix, such as (RO)_4_Si or (R’O)_3_SiR) [5,54]. In more detail, the first method, also known as grafting, can be developed using organosilanes, RSi(R’O)_3_, silazanes NH(SiR_3_)_3_ or chlorosilanes SiR_3_Cl reactions with free silanol groups present on the inorganic component surface [55]. This route shows advantages, such as the ability to retain the initial siliceous structure and the diminution of the porosity of the hybrid material, which depends on the size of the organic components. Nevertheless, the organic fractions are more evenly arranged in the simultaneous polymerization process of the inorganic and organic phase than in the grafting one [55]. In Table 1, the most recent studies on functionalized geopolymers are listed.

In the early 2000s, Hussain et al. reported the synthesis of new organic–inorganic hybrid systems obtained through the dispersion of geopolymers derived from kaolin treatment with potassium hydroxide into an epoxide matrix constituted by bisphenol, a diglycidyl ether (DGEBA). This study aimed to collect all the information to obtain better mechanical resistance and thermal behavior [69]. The results showed that the only addition of the precursor (kaolin) to the epoxide resin leads to an enhancement of the thermal stability, which is, in turn, further improved by the addition of the geopolymeric matrix to the DGEBA, thus demonstrating how these hybrid materials can act as flame retardants [69]. Another innovative approach for the design of new geopolymer hybrid materials, where an epoxy resin constitutes the organic moiety, was described by Ferone et al. [67]. They designed the novel material by incorporating the organic polymer in the geopolymeric matrix obtained from metakaolin as a precursor. The new synthetic method is based on the addition of an epoxy resin (obtained by mixing *N*,*N*-diglycidyl-4-glycidyl-oxyaniline with *bis*-(2-aminoethyl)amine or adding *N*,*N*-diglycidyl-4-glycidyl-oxyaniline to a mix of *bis*-(2-aminoethyl)amine and 2,4-diaminotoluene) to a geopolymeric suspension before the polymerization reactions of both reagents are completed, thus favoring an improvement of the affinity between the aqueous inorganic phases and the organic one. The results showed an enhancement of the mechanical properties, such as resistance to compressive strength and toughness concerning the pure non-functionalized geopolymer [67]. 

In addition, Colangelo et al. reported the synthesis and the characterization of metakaolin-based geopolymers functionalized with an epoxy resin [29]. These organic agents were added to the geopolymeric suspension in situ during the mixing process, allowing the formation of mortars with improved mechanical properties due to a reduced amount of internal crack and a more cohesive microstructure. The enhancement of the compressive strength, low thermal conductivity, and good fire resistance were observed also by Roviello et al., who designed new hybrid materials using a mixture of organic resins or dialkyl siloxane oligomers with metakaolin-based alkali-activated materials [28]. Fieset et al. recently developed an innovative method to synthesize new hybrid systems using thermosetting polymers to enhance the low toughness and compressive strength that characterizes high porous geopolymers [59]. In more detail, in the first step of their study, they designed geopolymer specimens with high open porosity by using hydrogen peroxide and saponified canola oil. In the second step, an orthophthalic polyester resin was intercalated into the porous structure with a consequent polymerization. This work provides a new opportunity to develop innovative ecological hybrid systems, using bioresins as organic agents and high porosity alkali-activated materials, whose mechanical and thermal properties would be scarce if used as neat geopolymers. As already mentioned, the addition of an organic agent to a geopolymer involves a structural modification of its inorganic matrix, leading to an improvement in the material mechanical properties. Lamanna et al. investigated the compressive strength of organic–inorganic materials obtained by the co-reticulation of a metakaolin-based geopolymer and polyethylene glycol (PEG) added in different ratios [68]. The different amounts of PEG significantly affect the material mechanical properties, leading to a very porous surface and internal cracks for products treated with higher PEG concentrations. Catauro et al. also performed a similar research work, through which they investigated the influence of both the aging time and the added PEG amount to the inorganic matrix [64]. FTIR analysis, performed by the authors, demonstrated the interaction between a metakaolin-based geopolymer and the polyethylene glycol by forming H bonds between the hydroxyl groups of the inorganic matrix and the PEG terminal alcoholic groups or its ethereal oxygen atoms. Furthermore, they reported an improvement in the inorganic network organization, with an increase in Al–O–Si bond formations over time, also affected by the amount of PEG with which the geopolymer matrix was treated [64]. At the same time, Yuana et al. proposed a similar hybrid material using polypropylene as an organic target, added in the powdered form. The use of electron microscopy allowed to demonstrate the formation of a crystalline microstructure on the innovative material surface, which acts as a reinforcing fiber, thus improving its mechanical properties [66]. As it is well known, different types of fiber, such as continuous or short ones, can be used to fortify composite materials and improve their toughness and strength by preventing the formation of structural cracks [70]. Recently, Guo et al. evaluated the mechanical properties and the sulfate resistance of different geopolymers functionalized with mineral and organic fibers, such as wollastonite and polypropylene [58]. The employment of both types of fibers in the same inorganic matrix enhanced the compressive strength concerning geopolymers reinforced using only organic fibers. Ramos et al. used polypropylene to develop new sustainable functionalized composite materials as well [57]. In particular, their study concerned the enhancement of geopolymer concrete waste by functionalizing this inorganic matrix with polypropylene and vinyl trimethoxysilane. The results reported by Ramos et al. showed an improvement in thermal stability and the elastic modulus. Through SEM analysis, the structural investigation observed an adhesion mechanism of polypropylene nanofilaments to the neat and functionalized inorganic matrix, which hinders the water incorporation. Organosilanes, as it is well known, play a key role in the functionalization of inorganic aluminosilicate-based systems. In recent years, the interest in new advanced engineering materials, which involve organosilane compounds, has considerably increased. Zhang et al. recently developed new hybrid materials, starting from metakaolin-based geopolymers, by using (3-aminopropyl)triethoxysilane (APTES) as a functionalizing and coupling agent [56]. This one is widely employed as a filling compound, surface coating protective material and a workability improver of mortars. The choice of using an organic compound containing an amino group rather than a simple alkoxysilane is due to the ability of the electron-rich nitrogen atom to form hydrogen bonds with the hydroxyl groups present in the inorganic matrix, leading to a further strengthening of the structure and a decrease in hydration of the composite paste [71]. Following the addition of APTES, the geopolymerization reaction is decelerated, leading to slow decomposition of the geopolymer lattice into the SiO_4_^4-^ and AlO_4_^5−^ tetrahedral, which recombine with the silane agent, forming a new network with greater structural homogeneity and higher compressive strength [56]. Dos Reis et al. provided new hybrid materials, starting from natural kaolin and some different silica-based organic compounds, such as APTES, tetraethylorthosilicate (TEOS), methylpolysiloxane and methyl phenyl polysiloxane, developed through the sol–gel method [61]. According to the SEM and TEM results, the functionalized materials showed better structural properties, which improved thermal, photophysical and photocatalytic features [61]. A similar study was carried out by Roviello et al., which synthesized new organic–inorganic materials, using fly ashes and a mixture of dimethylsiloxanes as precursors. In this case, the polymerization mechanism was the same as observed in the metakaolin-based hybrid compound synthesis, developed previously by the same authors. The organic units were embedded in the inorganic matrix [72]. The investigations on the mechanical properties provided satisfying data for the hybrid materials, with better results on tensile and compressive strength than the neat geopolymer. Furthermore, the synthetic method used in this case could be applied to design other organic–inorganic compounds, whose precursors could be other different types of aluminosilicate, such as blast furnace slag, muds or rice husk. The latter was employed together with fly ashes by Amritphale et al. to develop a novel synthetic approach for designing new sustainable materials [63]. The authors described a preparation method in which rice hulls were treated with a strongly alkaline solution, thus forming an organic–inorganic activator containing sodium silicate, D-glucose, sucrose and phenolic compounds present in the precursor. As already mentioned, organic polymers can improve the chemical, physical and mechanical properties of cement-based materials. In the last decades, in addition to studying the effects of organosilanes and organic polymers, such as polyethylene glycol and polyacrylamide, researchers also showed interest in polyacrylates to reshape the structure of geopolymers and improve their qualities. In this regard, Chen et al. described how polyacrylate affects the microstructure of geopolymers synthesized starting from metakaolin [60]. According to this study, the organic polymer incorporated in the inorganic matrix caused a reduction in the SiO_4_^4−^ polymerization degree, making more compact the geopolymeric paste and filling the pores, avoiding cracks. The hybrid material thus obtained showed higher flexural toughness. Other interesting polymers employed in geopolymer functionalizing processes are polyurethanes, thermoset compounds used in several industrial applications, such as a building. To the best of our knowledge, there is only a study in the literature about using this class of polymers as additives in the geopolymer synthesis process. The research work performed by Bergamonti et al. focused on studying the chemical, physical and mechanical features of geopolymers treated with polyurethane powders wastes [62]. FTIR investigations’ results allow classifying of the new organic–inorganic materials synthesized as I class compounds featured by weak interconnections. The data obtained on the flexural and compressive strengths demonstrate the better quality of the geopolymer product in terms of strength and compactness. Furthermore, thermal studies have shown that these new hybrid materials are able to isolate environments, thus representing an excellent green solution in the construction field. Thermal modification can be defined as chemical and physical changes produced on natural geopolymer matrices due to temperature. Several process variables have significant effects on properties [73]. The most important variables include the duration and temperature of treatment, the type of atmosphere, closed or open systems, sample size, and the use of catalysis. Most authors reported improvements in dimensional stability and reduced hygroscopicity of heat-treated natural geopolymer matrices. The heat treatments performed by the cement and geopolymer industry can be mainly classified as the following:

- Wetting and drying cycles refer to baking.
- Heat treatments.
- Hydrothermal process in which the fibers are heated in a liquid or vapor medium.

In addition, functionalized compounds can be employed as a valid alternative to the traditional materials in cultural heritage for restoration, consolidation and preservation of artworks. In this regard, Ouellet-Plamondon et al. experimented with a new way to synthesize new hybrid materials similar to Maya blue pigment [65]. This colorant is a nanohybrid material constituted by an organic moiety encapsulated in a palygorskite and sepiolite mineral [74]. In more detail, the authors synthesized a sepiolite/metakaolin-based geopolymer, functionalizing it with methylene blue or methyl red, focusing their attention on the sepiolite role as protector of the organic molecules encapsulated toward external agents. Physical interlocking and chemical bonds are the most relevant parameters displayed in geopolymer composites. Some studies have shown that the alkaline treatment provided a rough surface on the treated geopolymer matrices, thus facilitating the mechanical interlocking of the additives with the matrix, which in turn led to the improvement of the mechanical properties of the reinforced composites [75]. Regarding the chemical bonding mechanism, studies on silane-functionalized reinforced cementitious composites have suggested that silane coatings improve the adhesion of geopolymer components likely due to the polysiloxane network formed on the surface, resulting in a large number of active functional groups, which could chemically react with the matrix materials forming stable bonds. This study could be the starting point for developing new inorganic materials functionalized with organic dyes, which can be considered possible substitutes for the traditional binders sensitive to heat treatment in the production process of ordinary ceramic materials.

### 3.1. Functionalization by Sol–Gel Technique

The sol–gel technique is widely employed in developing hybrid geopolymeric based materials [5,76,77,78,79]. Moreover, as it is well known, this process is one of the primary approaches for manufacturing ceramic materials and glasses at low temperatures [80].

**Figure 7:**
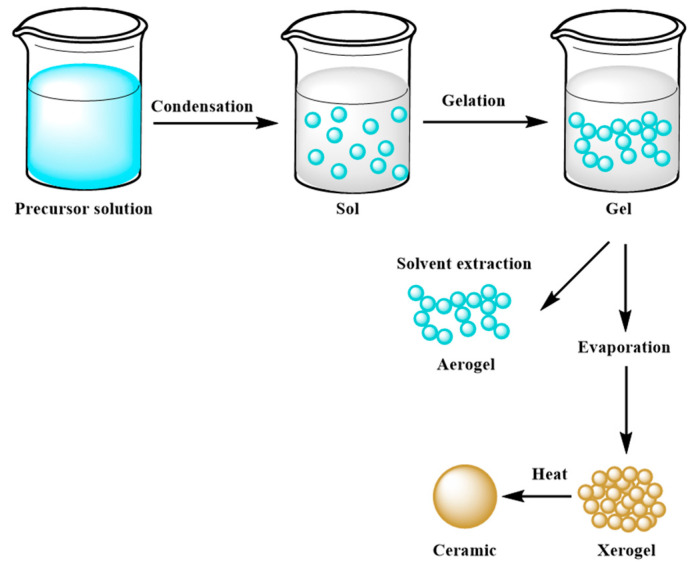
Scheme of the sol–gel mechanism.

The main goal of this method is to control the surface and interface of the designed materials during the first step of their production. It is also considered an effective coating process [81]. The most relevant feature of the sol–gel technique is the ease of homogeneously coating the entire surface of different materials, which find employment in various industrial fields, such as optical and sensing applications or powder synthesis of raw materials used as precursors in innovative concrete formulations [81,82,83,84,85,86,87]. Moreover, micro and mesoporous ceramics obtained through this method are widely employed in extraction technology, such as waste removal and drug delivery [88,89]. Nevertheless, sol–gel systems are affected by a low process rate, which can cause cracks inside the solidified gel [81]. Three ways allow to obtain a monolith from the sol–gel method: (a) gelation process starting from a colloidal solution; (b) hydrolysis and polycondensation reactions using alkoxides as precursors, such as Si(OR)_4_, where R is an alkyl chain, with hypercritical drying; (c) hydrolysis and polycondensation reactions, using alkoxides as precursors with drying at room temperature [90,91]. In more detail, the synthesis route of a monolith through this method consists in mixing the precursor materials in the liquid phase, with the consequent development of hydrolysis and polycondensation reactions, which lead to the formation of a low viscosity sol inside the solution. After that, a series of polycondensation reactions occur, forming a three-dimensional network structure gel with a continuous inorganic lattice containing an interconnected liquid phase, which will become a xerogel with a porous space structure after removal of the solvent by a drying process [87]. A schematic example of the sol–gel mechanism is shown in Figure 7.

In the case of alkali-activated cements, as well as geopolymers, as it is well known, the mechanical and chemical–physical properties are strongly influenced by the mineralogical composition of their aluminosilicate precursors. In a study by Chen et al., the effect of the NaOH precursor on the condensation mechanism in a geopolymeric matrix synthesized by the sol–gel methods was investigated. In particular, it was notable that gel particle size and polymerization degree increased with increasing alkalinity, whilst Si/Al ratio decreased [78]. Catauro et al. proposed a sol–gel synthesis route for Al_2_O_3_ · 2SiO_2_ powder as a precursor in geopolymer preparation [80]. Various samples differed from each other in the Si ratio and water content. The results showed a homogeneous Al-rich geopolymeric matrix for the molds with less water content. Moreover, a composite based on geopolymers synthesized through the sol–gel technique was designed by Magdaleno-Lòpez et al. The rice husk was added in the matrix, obtaining good results in terms of mechanical resistance and making it a material applied in the construction and restoration field [76]. An example of a sol–gel solution to be added to the geopolymer matrix contemplates the use of organosilane reagents. Silane coupling agents generally enhance the degree of crosslinking in composite materials. This treatment has been shown to improve the mechanical strength and water repellency of geopolymer composites. Zhou et al. modified bagasse fibers modified with alkyltrialkoxysilane or dialkyldialkoxysilane [92]. The silane solutions ranged from 0.5% to 8% by volume to increase their effectiveness. The researchers observed that bagasse fibers treated with 6% by volume of silane solution improved the size and porosity of the fibers, thereby decreasing the water absorption and setting time of the cementitious composites. Finally, this synthesis method was applied to the design, development and study of geopolymer-based coatings with flame-retardant properties, starting from blast furnace slag and fly ash used as precursor materials [93].

### 3.2. Functionalization with Nanoparticles (NPs)

Recent studies showed that the addition of nanoparticles to the geopolymer matrix improves the chemical–physical, mechanical and structural characteristics of the alkaline-activated products. Nanomaterials can create intelligent materials with improved rheological and mechanical properties, thanks to their small size and high surface-to-volume ratio [94]. The addition of these agents, such as nano-silica, nano alumina, TiO_2_, ZnO and graphene oxide, plays a key role in the consolidation of the innovative material, acting as nanofillers, and leading to a reduction of the inner water together with an increase in the compressive strength [95,96,97,98,99,100]. Recently, a study investigated the effect of a geopolymer treated with Al_2_O_3_ and multi-walled carbon nanotubes (MWCNT) functionalized with hydroxyl groups [94]. 

**Figure 8:**
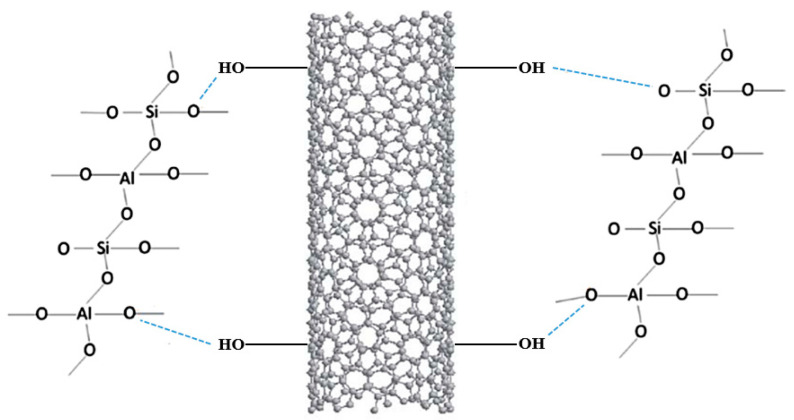
Scheme of the sol–gel mechanism.

The results reported by Alvi et al. showed an enhancement of the mechanical properties in the treated product compared to the neat one, as the addition of these nanomaterials provided a more malleable structure with higher compressive and tensile strength [94]. Furthermore, as reported in Figure 8, the functionalization of the material under study was possible, thanks to forming a hydrogen bond between the hydroxyl group of the MWCNT and the oxygen atoms of the geopolymer 3D structure. 

Kumar and Yuvaraj also described a research work in which carbon nanotubes were studied as fillers of geopolymeric concrete, underlining how these nanomaterials can increase their durability over time by preventing the formation of cracks in concrete and ceramic bodies [101]. In addition, Halloysite nanotubes also presented consolidating properties in polymeric matrices so that they can be studied as possible reinforcing agents in the field of cultural heritage for those artworks or finds deteriorated by aging [102]. 

As recently reported in the literature, incorporating nano-sized graphene into the geopolymeric paste enhanced the resistance and the hardness of these green concretes [99,101]. This improvement in the mechanical properties is probably due to several factors, such as the concentration of the alkaline solution, the type of graphene and the surface graphene changes that occur after the treatment with basic solutions. Moreover, these nanoparticles, aging as fillers, occupying the voids in the polymeric porous structure, make the material more compact and resistant. From a chemical point of view, using an alkaline solution causes the reduction of graphene oxide, providing an epoxy group, which can weakly interact with the geopolymeric structure, dispersing in the matrix more efficiently [99].

In the last decades, TiO_2_ nanoparticles have been employed as additive agents in concretes, paints, tiles and antifogging coatings, also showing photocatalytic properties for the protection from UV light degradation [103]. In a paper reported by Maiti et al., the integration of TiO_2_ nanoparticles of 30 nm in a fly ash-based geopolymeric matrix led to a more compact and densified formulation with a decrease of the porosity and the roughness of the structure, thus enhancing mechanical properties [98]. Moreover, the specimens under study showed a low water absorption and a good resistance to photodegradation. A significant improvement in the hardness and physical–chemical features of a similar materials was described by Kantarci and Maraş, which develop a nano TiO_2_-modified geopolymer mortar to be applied in damaged beam–column joints [104]

The same behavior toward the ultraviolet irradiation was found in geopolymers treated with ZnO or Cu_2_O nanoparticles [97,105]. In a work presented by Zidi et al., the nano-size ZnO incorporation in a geopolymeric matrix enhanced the mechanical and the thermal properties of the mortar under study, showing a compressive strength comparable to the Portland cement one [106]. Instead, in a work reported by Siyal et al., a metakaolin-based geopolymer functionalized with cuprous oxide nanoparticles showed the ability to absorb a methylene blue dye widely used in the field of cultural heritage, both in the dark and in the presence of UV radiation, due to an increase in the size of the pores inside the geopolymeric matrix [105]. On the contrary, nanosilver-geopolymers can be applied to protect wall surfaces in constructions, artworks and other finds, preventing the growth of bacteria and the formation of biofilm, which can lead to degradation [107]. Nevertheless, the best results in terms of functionalization were obtained by adding SiO_2_ silica nanoparticles to the cement paste, as they are the main constituent of the geopolymeric matrix. This is due to a stronger interaction toward the functional groups of the precursors used to synthesize the mortar. In a paper presented by Rahmawati et al., the treatment of the cement paste with nanosilica enhanced the anticorrosive properties to the sulfate, and the further addition of cellulose nanoparticles strengthened the concrete, as they can fill the voids present in the matrix [96]. Although recently in the literature, the studies that reported on the different applications of geopolymers functionalized with several nanomaterials appear encouraging in terms of mechanical, photocatalytic and antifouling properties, there are still great difficulties in transferring this type of technology on a large scale.

## 4. Applications in Construction Industry and Cultural Heritage Fields

Geopolymers exhibit structure and mineralogical properties very similar to ceramic bodies. Their chemical, physical and mechanical characteristics, which make them highly durable over time, allow them to be used as fillers for voids and cracks in tiles and repairing mortars as an alternative to traditional materials in cultural heritage. These sustainable binders are featured by some peculiarities that make them potentially applicable in the field of conservation and restoration, such as robustness in extreme environmental conditions, adhesion to the ceramic substrate facilitated by the similar structure, and chemical–physical properties fast drying and polymerization [108]. Nevertheless, they also have drawbacks, such as the corrosion of the historical enamel of an artwork or the formation of soluble salts when the material is allowed to dry in the air, often due to the high concentration of the alkaline activator used in the reaction of geopolymerization, which leads to carbonation processes. The use of geopolymers and hybrid materials deriving from these green binders in the field of cultural heritage is still the subject of study for many researchers. However, several investigations on the conservation of stones for repairing tiles and consolidating architectural materials in the last decades have been conducted [6,109]. Geraldes et al., for example, discussed the possibility of using metakaolin-based geopolymers in tile reparation, filling the lesions with the innovative mortar in the ceramic body, and confirmed that the evaporation of water represents a crucial role in the polymerization reaction, as it affects the cracking and the adhesion mechanism of the geopolymer paste to the ceramic [6]. The chance of developing sustainable materials to restore architectonical and building structures was recently discussed by Moutinho et al., who studied the possibility of using geopolymers obtained from commercial metakaolin as a repair material for filling cracks in Portuguese tiles [110]. Similar research work was conducted by Clausi et al. and Baltazar et al. [109,111]. In more detail, the first ones evaluated the possibility of creating new green binders always starting from the metakaolin and mixing it with aggregates deriving from ornamental stones, such as Pietra Serena and Pietra di Angera [111]. Nevertheless, the results showed a reduction in mechanical strength compared to that observed for a neat geopolymer, which, however, falls within the standards of mortars. For this reason, this new material can be used for the restoration of stone objects. Moreover, Clausi et al. evaluated the possibility of using a geopolymer mortar with rock particles to obtain binders that showed good aesthetic compatibility with stone buildings [111]. Instead, Baltazar et al. designed new geopolymers starting from fly ashes and assessed the possibility of using these as consolidant materials in old stone constructions [109]. Thus, in addition to the simple use of neat geopolymers as substitutes for traditional mortars in the field of cultural heritage restoration, it is possible to develop new materials, as previously mentioned, which are functionalized with molecules of a different nature, to improve their mechanical, chemical, physical and aesthetic properties. In light of this consideration, just as Ricciotti et al. developed new functionalized hybrid materials, using epoxy resins in addition to neat metakaolin-based geopolymers, the authors of this review designed a possible synthesis method to obtain new implemented green binders [5,26]. The studies have highlighted the high compatibility of the geopolymeric binder with different materials and the advantage of low CO_2_ emissions, the use of industrial waste by-products in their production, and resistance to atmospheric pollution and aggressive agents. By exploiting the combination of different alkaline precursors and activators, geopolymers can achieve competitive mechanical properties and significant environmental benefits. With specially designed formulations, the materials can be fireproof, breathable, and resistant to rising salts and acid rain. In addition, an added benefit is the ability to imitate natural, manufactured, and stone materials. There are many articles on the characteristics and properties of both precursors and the final product, but only a few concerns the application of cultural heritage. Despite this, the data reported by the few publications present today suggest the use of these materials for the consolidation, conservation, and restoration of the heritage built inside historical centers, where the low CO_2_ emissions and the characteristics shown by the geopolymers could bring an enormous benefit to the environment and the protection of the structures themselves [112].

## 5. Conclusions

In the last decades, geopolymer mortars have captured the attention of an increasing number of researchers because of their particular features and potential application in many industrial fields, particularly as a good sustainable alternative to cement. In particular, since the development of unconventional and eco-friendly materials in substitution of the Portland cement has become a crucial research line worldwide, geopolymers production seem to play a crucial role in order to reduce the pollution of gas emissions and provide significant energy savings. Nevertheless, although the growth made on these new alternative materials has improved in recent times, thanks to their potential employment in many applications and to their different advantages (i.e., sustainability, simple synthetic procedures, and improvement of their surface properties by proper functionalization techniques), their performance and the factors affecting them are still unclear.

This review is intended to provide a comprehensive summary starting from these alkaline-activated materials to their hybrid compounds functionalized with organic compounds, polymeric matrices and nanoparticles, focusing on purposes in the cultural heritage field and building application fields. In particular, focusing initially on the development, the composition, the polymerization reaction mechanism and the influencing factors of performance of the developed geopolymers, the paper offers an overview of new functional geopolymeric matrix and hybrid sol–gel or opportune nanomaterials used to improve their potential hydrophobic, anti-abrasive, antibacterial and antifouling properties. Pre-treatment methods are aimed at improving the mechanical and physical properties of the geopolymer; the matrices mentioned above mainly include chemical agents, such as sodium or potassium hydroxide (NaOH, KOH), sodium silicate (Na_2_SiO_3_), silane and alkoxysilane agents, and metal nanoparticles. Furthermore, the combination of matrix pre-treatments, such as hardening before polymer coating, show synergistic effects on cementitious composites. The reinforced geopolymer materials generally show promising structural performance, such as interface strength, thermal stability, and improved mechanical properties.

Moreover, the influence of the alkaline agent in terms of concentration and the different raw materials used as precursors on the properties of the geopolymer, such as compressive strength and hardness of the synthesis product obtained, is also discussed. In fact, a key role in the preparation of geopolymer binders is played by the nature and the dosage of alkali activators, which show a significant effect on the hydration, microstructure, and performance of these new mortars, and still represent the only non-sustainable factor and disadvantage in the geopolymerization process. However, further studies on their chemical and physical properties are needed to develop materials with greater mechanical strength and longer life.

## Figures and Tables

**Table 1:** Organic–inorganic hybrid geopolymers present in literature.

Inorganic Substrate	Organic Agent	Ref.
Metakaolin-based geopolymer	(3-Aminopropyl)triethoxysilane (APTES)	[56]
Geopolymer concrete waste	Vinyl trimethoxy silane (VTP) + recycled polypropylene (rPP)	[57]
Metakaolin-based geopolymer	Polypropylene fiber (PP), polyvinyl alcohol fiber (PVA)	[58]
Metakaolin-based geopolymer	Unsaturated orthophtalic polyester resin	[59]
Metakaolin-based geopolymer	polyacrylate	[60]
Fly ash- based geopolymer	Oligomeric dimethylsiloxane	[54]
Kaolin-based geopolymer	Methyl-polysiloxane (MK), methyl-phenyl-polysiloxane (H44), tetraethyl-ortho-silicate (TEOS) and 3-amino-propyl-triethoxysilane (APTES)	[61]
Metakaolin-based geopolymer	Polyurethane powders wastes (polyurethane foam and polyisocyanurate foam)	[62]
Metakaolin-based geopolymer	Commercial oligomeric dimethylsiloxane mixture and epoxy resin	[28]
Fly ash-based geopolymer	Organic molecules deriving from the decomposition of rice husk: D-glucose, native cellulose, phenolic compounds and sucrose	[63]
Metakaolin-based geopolymer	Polyethylene glycol (PEG)	[64]
Metakaolin-based geopolymer + sepiolite	Methylene blue (MB) and methyl red (MR)	[65]
Metakaolin-based geopolymer	Polyethylene (PE)	[66]
Metakaolin-based geopolymer	Commercial epoxy resin	[29]
Metakaolin-based geopolymer	Epoxy resins formed by *N*,*N*-diglycidyl-4-glycidyl-oxyaniline with bis-(2-aminoethyl)amine and *N*,*N*-diglycidyl-4-glycidyl-oxyaniline with bis-(2-aminoethyl)amine and 2,4-diaminotoluene)	[67]
Metakaolin-based geopolymer	Polyethylene glycol (PEG)	[68]
Kaolin-based geopolymer	Epoxide matrix constituted by bisphenol a diglycidyl ether	[69]

## References

[1] Ozga I., Ghedini N., Giosuè C., Sabbioni C., Tittarelli F., Bonazza A. (2014). Assessment of air pollutant sources in the deposit on monuments by multivariate analysis. Sci. Total Environ..

[2] Hanzlíček T., Steinerová M., Straka P., Perná I., Siegl P., Švarcová T. (2009). Reinforcement of the terracotta sculpture by geopolymer composite. Mater. Des..

[3] Occhipinti R., Stroscio A., Finocchiaro C., Fugazzotto M., Leonelli C., José Lo Faro M., Megna B., Barone G., Mazzoleni P. (2020). Alkali activated materials using pumice from the Aeolian Islands (Sicily, Italy) and their potentiality for cultural heritage applications: Preliminary study. Constr. Build. Mater..

[4] Valluzzi M.R., Modena C., de Felice G. (2014). Current practice and open issues in strengthening historical buildings with composites. Mater. Struct..

[5] Ielo I., Galletta M., Rando G., Sfameni S., Cardiano P., Sabatino G., Drommi D., Rosace G., Plutino M. (2020). Design, synthesis and characterization of hybrid coatings suitable for geopolymeric-based supports for the restoration of cultural heritage. IOP Conf. Ser. Mater. Sci. Eng..

[6] Geraldes C.F.M., Lima A.M., Delgado-Rodrigues J., Mimoso J.M., Pereira S.R.M. (2016). Geopolymers as potential repair material in tiles conservation. Appl. Phys. A.

[7] Duxson P., Fernández-Jiménez A., Provis J.L., Lukey G.C., Palomo A., van Deventer J.S.J. (2007). Geopolymer technology: The current state of the art. J. Mater. Sci..

[8] Davidovits J. (2017). Geopolymers: Ceramic-like inorganic polymers. J. Ceram. Sci. Technol..

[9] Nodehi M., Taghvaee V.M. (2021). Alkali-Activated Materials and Geopolymer: A Review of Common Precursors and Activators Addressing Circular Economy. Circ. Econ. Sustain..

[10] Obonyo E.A., Kamseu E., Lemougna P.N., Tchamba A.B., Melo U.C., Leonelli C. (2014). A Sustainable Approach for the Geopolymerization of Natural Iron-Rich Aluminosilicate Materials. Sustainability.

[11] Kaze R.C., à Moungam L.M.B., Djouka M.L.F., Nana A., Kamseu E., Melo U.F.C., Leonelli C. (2017). The corrosion of kaolinite by iron minerals and the effects on geopolymerization. Appl. Clay Sci..

[12] Jihui Z. (2021). Eco-friendly geopolymer materials: A review of performance improvement, potential application and sustainability assessment. J. Clean. Prod..

[13] Davidovits J. (2020). Geopolymer Chemistry and Applications.

[14] Provis J.L., Duxson P., van Deventer J.S.J. (2010). The role of particle technology in developing sustainable construction materials. Adv. Powder Technol..

[15] Bakhtyar B., Kacemi T., Nawaz M.A. (2017). A Review on Carbon Emissions in Malaysian Cement Industry. Int. J. Energy Econ. Policy.

[16] Provis J.L., van Deventer J.S.J. (2009). Geopolymers: Structures, Processing, Properties and Industrial Applications.

[17] Glukhovsky V.D. (1959). Soil Silicates (Gruntosilikaty).

[18] Davidovits J. (1991). Geopolymers—Inorganic polymeric new materials. J. Therm. Anal..

[19] Shi C., Roy D., Pavel K. (2006). Alkaline Activators. Alkali Activated Cenents & Concrete.

[20] Shi C., Krivenko P.V., Roy D.M. (2006). Alkali-Activated Cements and Concretes.

[21] Silverstrim T., Rostami H., Larralde J., Samadi A. (1997). Fly Ash Cementitious Material and Method of Making A Product. U.S. Patent.

[22] Palomo A., Grutzeck M.W., Blanco M.T. (1999). Alkali-activated fly ashes—A cement for the future. Cem. Concr. Res..

[23] Duxson P., Provis J.L. (2008). Designing Precursors for Geopolymer Cements. J. Am. Ceram. Soc..

[24] Kaze C.R., Nana A., Lecomte-Nana G.L., Deutou J.G.N., Kamseu E., Melo U.C., Andreola F., Leonelli C. (2021). Thermal behaviour and microstructural evolution of metakaolin and meta-halloysite-based geopolymer binders: A comparative study. J. Therm. Anal. Calorim..

[25] Ekinci E., Kantarci F., Karakoc M.B., Turkmen I. Effect of silica modulus on compressive strength of volcanic tuff based geopolymer concrete. Proceedings of the 185th IIER International Conference.

[26] Ricciotti L., Molino A.J., Roviello V., Chianese E., Cennamo P., Roviello G. (2017). Geopolymer Composites for Potential Applications in Cultural Heritage. Environments.

[27] Laskar A.I., Talukdar S. (2008). Rheological behavior of high performance concrete with mineral admixtures and their blending. Constr. Build. Mater..

[28] Roviello G., Menna C., Tarallo O., Ricciotti L., Messina F., Ferone C., Asprone D., Cioffi R. (2017). Lightweight geopolymer-based hybrid materials. Compos. Part B Eng..

[29] Colangelo F., Roviello G., Ricciotti L., Ferone C., Cioffi R. (2013). Preparation and Characterization of New Geopolymer-Epoxy Resin Hybrid Mortars. Materials.

[30] Trovato V., Rosace G., Colleoni C., Sfameni S., Migani V., Plutino M. (2020). Sol-gel based coatings for the protection of cultural heritage textiles. IOP Conf. Ser. Mater. Sci. Eng..

[31] Nikolova A., Rostovskya I., Nugterenb H. (2017). Geopolymer materials based on natural zeolite. Case Stud. Constr. Mater..

[32] Barbosa V.F.F., MacKenzie K.J.D., Thaumaturgoa C. (2000). Synthesis and characterisation of materials based on inorganic polymers of alumina and silica: Sodium polysialate polymers. Int. J. Inorg. Mater..

[33] Rowles M.R., Hanna J.V., Pike K.J., Smith M.E. (2007). ^29^Si, ^27^Al, ^1^H and ^23^Na MAS NMR Study of the Bonding Character in Aluminosilicate Inorganic Polymers. Appl. Magn. Reson..

[34] Škvára F. (2007). Alkali activated materials or geopolymers?. Ceramics−Silikáty.

[35] Criado M., Fernández-Jiménez A., Palomo A. (2007). Alkali activation of fly ash: Effect of the SiO_2_/Na_2_O ratio: Part I: FTIR study. Microporous Mesoporous Mater..

[36] Torres-Carrasco M., Puertas F. (2017). Alkaline activation of aluminosilicates as an alternative to Portland cement: A Review. Rom. J. Mater..

[37] Khale D., Chaudhary R. (2007). Mechanism of geopolymerization and factors influencing its development: A review. J. Mater. Sci..

[38] Glukhovsky V.D., Rostovkaya G.S., Rumyna G.V. High strength slag-alkali cement. Proceedings of the 7th International Congress on the Chemistry of Cements.

[39] Pacheco Torgal F., Labrincha J.A., Leonelli C., Palomo A., Chindaprasirt P. (2015). Handbook of Alkali-Activated Cements, Mortars and Concretes.

[40] Garcí T.A., de Lourdes Chávez-Garcí M. (2016). Compressive Strength of Metakaolin-Based Geopolymers: Influence of KOH Concentration, Temperature, Time and Relative Humidity. Mater. Sci. Appl..

[41] Shi C., Day R.L. (2000). Pozzolanic reactions in the presence of chemical activators—Part II: Reaction mechanisms. Cem. Concr. Res..

[42] Błaszczyński T.Z., Król M.R. (2017). Alkaline Activator Impact on the Geopolymer Binders. IOP Conf. Ser. Mater. Sci. Eng..

[43] Palomo A., Alonso S., Fernández-Jiménez A., Sobrados I., Sanz J. (2004). Alkali activated of fly ashes, A NMR study of the reaction products. J. Am. Ceram. Soc..

[44] Brouwers H.J.H., Van Eijk R.J. (2002). Reactivity of fly ash: Extension and application of a shrinking core model. Concr. Sci. Eng..

[45] Van Jaarsveld J.G.S., Van Deventer J.S.J. (1989). Effect of alkali-metal activator on the properties of fly ash-based geopolymers. Ind. Eng. Chem. Res..

[46] Runzhang Y., Shi-Zi O., Qiong-Ying G. (1987). Study on structure an latent hydraulic activity of slag and its activation mechanism. J. Wuhan Univ. Technol..

[47] Jiao Z., Wang Y., Zheng W., Huang W. (2018). Effect of Dosage of Alkaline Activator on the Properties of Alkali-Activated Slag Pastes. Adv. Mater. Sci. Eng..

[48] Song S., Sohn D., Jennings H.M., Mason T.O. (2000). Hydration of alkaliactivated ground granulated blast furnace slag. J. Mater. Sci..

[49] Shi C., Li Y., Tang X. (1989). A preliminary investigation on the activation mechanism of granulated phosphorus slag. J. Southeast Univ. Nanjing PR China.

[50] Joshi S.V., Kadu M.S. (2012). Role of Alkaline Activator in Development of Eco-friendly Fly Ash Based Geo Polymer Concrete. Int. J. Environ. Sci. Dev..

[51] Neville A.M. (2011). Properties of Concrete.

[52] Kresse P. (1991). Efflorescence and its prevention. Betonw. Fert.-Tech..

[53] Faustini M., Nicole L., Ruiz-Hitzky E., Sanchez C. (2018). History of Organic–Inorganic Hybrid Materials: Prehistory, Art, Science, and Advanced Applications. Adv. Funct. Mater..

[54] Roviello G., Ricciotti L., Molino A.J., Menna C., Ferone C., Cioffi R., Tarallo O. (2019). Hybrid Geopolymers from Fly Ash and Polysiloxanes. Molecules.

[55] Hoffmann F., Cornelius M., Morell J., Fröba M. (2006). Silica-Based Mesoporous Organic–Inorganic Hybrid Materials. Angew. Chem. Int. Ed..

[56] Zhang C., Hu Z., Zhu H., Wang X., Gao J. (2020). Effects of silane on reaction process and microstructure of metakaolin-based geopolymer composites. J. Build. Eng..

[57] Ramos F.J.H.T.V., da Silva M.H.P., Monteiro S.N., Grafov A., Grafova I. (2020). Recycled polypropylene matrix nanocomposites reinforced with silane functionalized geopolymer concrete waste. J. Mater. Res. Technol..

[58] Guo L., Wu Y., Xu F., Song X., Ye J., Duan P., Zhang Z. (2020). Sulfate resistance of hybrid fiber reinforced metakaolin geopolymer composites. Compos. Part B Eng..

[59] Fiset J., Cellier M., Vuillaume P.Y. (2020). Macroporous geopolymers designed for facile polymers post-infusion. Cem. Concr. Compos..

[60] Chen X., Zhou M., Ge X., Niu Z., Guo Y. (2019). Study on the microstructure of metakaolin-based geopolymer enhanced by polyacrylate. J. Ceram. Soc. Jpn..

[61] dos Reis G.S., Lima E.C., Sampaio C.H., Rodembusch F.S., Petter C.O., Cazacliu B.G., Dotto G.L., Hidalgo G.E.N. (2018). Novel kaolin/polysiloxane based organic-inorganic hybrid materials: Sol-gel synthesis, characterization and photocatalytic properties. J. Solid State Chem..

[62] Bergamonti L., Taurino R., Cattani L., Ferretti D., Bondioli F. (2018). Lightweight hybrid organic-inorganic geopolymers obtained using polyurethane waste. Constr. Build. Mater..

[63] Amritphale S.S., Mishra D., Mudgal M., Chouhan R.K., Chandra N. (2016). A novel green approach for making hybrid inorganic- organic geopolymeric cementitious material utilizing fly ash and rice husk. J. Environ. Chem. Eng..

[64] Catauro M., Papale F., Lamanna G., Bollino F. (2015). Geopolymer/PEG Hybrid Materials Synthesis and Investigation of the Polymer Influence on Microstructure and Mechanical Behavior. Mater. Res..

[65] Ouellet-Plamondon C., Aranda P., Favier A., Habert G., van Damme H., Ruiz-Hitzky E. (2015). The Maya blue nanostructured material concept applied to colouring geopolymers. RSC Adv..

[66] Yuan X.W., Easteal A.J., Bhattacharyya D. (2004). Geopolymer Reinforced Polyethylene Nanocomposites. Compos. Technol. For. 2020.

[67] Ferone C., Roviello G., Colangelo F., Cioffi R., Tarallo O. (2013). Novel hybrid organic-geopolymer materials. Appl. Clay Sci..

[68] Lamanna G., Soprano A., Bollino F., Catauro M. (2013). Mechanical Characterization of Hybrid (Organic-Inorganic) Geopolymers. Key Eng. Mater..

[69] Hussain M., Varely R., Cheng Y.B., Mathys Z., Simon G.P. (2005). Synthesis and thermal behavior of inorganic–organic hybrid geopolymer composites. J. Appl. Polym. Sci..

[70] Shaikh F.U.A. (2013). Review of mechanical properties of short fibre reinforced geopolymer composites. Constr. Build. Mater..

[71] Švegl F., Šuput-Strupi J., Škrlep L., Kalcher K. (2008). The influence of aminosilanes on macroscopic properties of cement paste. Cem. Concr. Res..

[72] Roviello G., Menna C., Tarallo O., Ricciotti L., Ferone C., Colangelo F., Asprone D., di Maggio R., Cappelletto E., Prota A. (2015). Preparation, structure and properties of hybrid materials based on geopolymers and polysiloxanes. Mater. Des..

[73] Kaewkuk S., Sutapun W., Jarukumjorn K. (2013). Effects of interfacial modification and fiber content on physical properties of sisal fiber/polypropylene composites. Compos. Part B Eng..

[74] Nicole L., Laberty-Robert C., Rozes L., Sanchez C. (2014). Hybrid materials science: A promised land for the integrative design of multifunctional materials. Nanoscale.

[75] Camargo M.M., Adefrs Taye E., Roether J.A., Tilahun Redda D., Boccaccini A.R. (2020). A Review on Natural Fiber-Reinforced Geopolymer and Cement-Based Composites. Materials.

[76] Magdaleno-López C., Pérez-Bueno J.d.J., Flores-Segura J.C., Reyes-Araiza J.L., Mendoza-López M.L., Arés O., Manzano-Ramírez A. (2018). A geopolymeric composite of non-calcined rice husks made of metakaolin/sol–gel silica. J. Compos. Mater..

[77] Catauro M., Bollino F., Dell’Era A., Ciprioti S.V. (2016). Pure Al_2_O_3_·2SiO_2_ synthesized via a sol-gel technique as a raw material to replace metakaolin: Chemical and structural characterization and thermal behavior. Ceram. Int..

[78] Chen X., Mondal P. (2020). Effects of NaOH amount on condensation mechanism to form aluminosilicate, case study of geopolymer gel synthesized via sol–gel method. J. Sol. Gel. Sci. Technol..

[79] Huang B., Li C., Zhang Y., Ding W., Yang M., Yang Y., Zhai H., Xu X., Wang D., Debnath S. (2021). Advances in fabrication of ceramic corundum abrasives based on sol–gel process. Chin. J. Aeronaut..

[80] Catauro M., Bollino F., Cattaneo A.S., Mustarelli P. (2017). Al_2_O_3_·2SiO_2_ powders synthesized via sol–gel as pure raw material in geopolymer preparation. J. Am. Ceram. Soc..

[81] Dehghanghadikolaei A., Ansary J., Ghoreishi R. (2018). Sol-Gel Process Applications: A mini-Review. Proc. Nat. Res. Soc..

[82] Plutino M.R., Guido E., Colleoni C., Rosace G. (2017). Effect of GPTMS functionalization on the improvement of the pH-sensitive methyl red photostability. Sens. Actuators B Chem..

[83] Rosace G., Guido E., Colleoni C., Brucale M., Piperopoulos E., Milone C., Plutino M.R. (2017). Halochromic resorufin-GPTMS hybrid sol-gel: Chemical-physical properties and use as pH sensor fabric coating. Sens. Actuators B Chem..

[84] Guido E., Colleoni C., De Clerck K., Plutino M.R., Rosace G. (2014). Influence of catalyst in the synthesis of a cellulose-based sensor: Kinetic study of 3-glycidoxypropyltrimethoxysilane epoxy ring opening by Lewis acid. Sens. Actuators B Chem..

[85] Trovato V., Colleoni C., Castellano A., Plutino M.R. (2018). The key role of 3-glycidoxypropyltrimethoxysilane sol–gel precursor in the development of wearable sensors for health monitoring. J. Sol. Gel. Sci. Technol..

[86] Rosace G., Trovato V., Colleoni C., Caldara M., Re V., Brucale M., Piperopoulos E., Mastronardo E., Milone C., De Luca G. (2017). Structural and morphological characterizations of MWCNTs hybrid coating onto cotton fabric as potential humidity and temperature wearable sensor. Sens. Actuators B Chem..

[87] Ielo I., Giacobello F., Sfameni S., Rando G., Galletta M., Trovato V., Rosace G., Plutino M.R. (2021). Nanostructured Surface Finishing and Coatings: Functional Properties and Applications. Materials.

[88] Puoci F., Saturnino C., Trovato V., Iacopetta D., Piperopoulos E., Triolo C., Bonomo M.G., Drommi D., Parisi O.I., Milone C. (2020). Sol–Gel Treatment of Textiles for the Entrapping of an Antioxidant/Anti-Inflammatory Molecule: Functional Coating Morphological Characterization and Drug Release Evaluation. Appl. Sci..

[89] Ren Z., Zhu X., Du J., Kong D., Wang N., Wang Z., Wang Q., Liu W., Li Q., Zhou Z. (2018). Facile and green preparation of novel adsorption materials by combining sol-gel with ion imprinting technology for selective removal of Cu(II) ions from aqueous solution. Appl. Surf. Sci..

[90] Tan W.K., Muto H., Kawamura G., Lockman Z., Matsuda A. (2021). Nanomaterial Fabrication through the Modification of Sol–Gel Derived Coatings. Nanomaterials.

[91] Kessler V.G., Klein L., Aparicio M., Jitianu A. (2018). The Synthesis and Solution Stability of Alkoxide Precursors. Handbook of Sol-Gel Science and Technology: Processing, Characterization and Applications.

[92] Zhou F., Cheng G., Jiang B. (2014). Effect of silane treatment on microstructure of sisal fibers. Appl. Surf. Sci..

[93] Wang Y., Zhao J. (2019). Facile preparation of slag or fly ash geopolymer composite coatings with flame resistance. Constr. Build. Mater..

[94] Alvi M.A.A., Khalifeh M., Agonafir M.B. (2020). Effect of nanoparticles on properties of geopolymers designed for well cementing applications. J. Pet. Sci. Eng..

[95] Rashad A.M. (2019). Effect of nanoparticles on the properties of geopolymer materials. Mag. Concr. Res..

[96] Rahmawati C., Aprilia S., Saidi T., Aulia T.B. (2021). Current development of geopolymer cement with nanosilica and cellulose nanocrystals. J. Phys. Conf. Ser..

[97] Guzmán-Carrillo H.R., Manzano-Ramírez A., Garcia Lodeiro I., Fernández-Jiménez A. (2020). ZnO Nanoparticles for Photocatalytic Application in Alkali-Activated Materials. Molecules.

[98] Maiti M., Sarkar M., Maiti S., Malik M.A., Xu S. (2020). Modification of geopolymer with size controlled TiO_2_ nanoparticle for enhanced durability and catalytic dye degradation under UV light. J. Clean. Prod..

[99] Tay C.H., Norkhairunnisa M. (2021). Mechanical Strength of Graphene Reinforced Geopolymer Nanocomposites: A Review. Front. Mater..

[100] Shehata N., Sayed E.T., Abdelkareem M.A. (2021). Recent progress in environmentally friendly geopolymers: A review. Sci. Total Environ..

[101] Kumar M.M., Yuvaraj S. (2020). Behaviour of Different Nanomaterials in Geopolymer Concrete. Int. J. Innov. Technol. Explor. Eng..

[102] Cavallaro G., Milioto S., Lazzara G. (2020). Halloysite Nanotubes: Interfacial Properties and Applications in Cultural Heritage. Langmuir.

[103] Janczarek M., Klapiszewski Ł., Jędrzejczak P., Klapiszewska I., Ślosarczyk A., Jesionowski T. (2021). Progress of functionalized TiO_2_-based nanomaterials in the construction industry: A comprehensive review. Chem. Eng. J..

[104] Kantarcı F., Maraş M.M. (2022). Formulation of a novel nano TiO_2_-modified geopolymer grout for application in damaged beam-column joints. Constr. Build. Mater..

[105] Siyal A.A., Shamsuddin M.R., Khan M.I., Rabat N.E., Zulfiqar M., Man Z., Siame J., Azizli K.A. (2018). A review on geopolymers as emerging materials for the adsorption of heavy metals and dyes. J. Environ. Manag..

[106] Zidi Z., Ltifi M., Ayadi Z.B., Mir L.E., Nóvoa X.R. (2020). Effect of nano-ZnO on mechanical and thermal properties of geopolymer. J. Asian Ceram. Soc..

[107] Armayani M., Pratama M.A. (2017). Subaer The Properties of Nano Silver (Ag)-Geopolymer as Antibacterial Composite for Functional Surface Materials. MATEC Web Conf..

[108] Kürklü G., Görhan G. (2019). Investigation of usability of quarry dust waste in fly ash-based geopolymer adhesive mortar production. Constr. Build. Mater..

[109] Baltazar L.G., Henriques F.M.A., Temporão D., Cidade M.T. (2019). Experimental Assessment of Geopolymer Grouts for Stone Masonry Strengthening. Key Eng. Mater..

[110] Moutinho S., Costa C., Andrejkovičová S., Mariz L., Sequeira C., Terroso D., Rocha F., Velosa A. (2020). Assessment of properties of metakaolin-based geopolymers applied in the conservation of tile facades. Constr. Build. Mater..

[111] Clausi M., Tarantino S.C., Magnani L.L., Riccardi M.P., Tedeschi C., Zema M. (2016). Metakaolin as a precursor of materials for applications in Cultural Heritage: Geopolymer-based mortars with ornamental stone aggregates. Appl. Clay Sci..

[112] Pagnotta S., Tenorio A., Tiné M., Lezzerini M. Geopolymers as a potential material for preservation and restoration of Urban Build Heritage: An overview. Proceedings of the IOP Conference Series: Earth and Environmental Science, 6th World Multidisciplinary Earth Sciences Symposium.

